# Supplementary material for: Evaluating Completeness of Foodborne Outbreak Reporting in the United States, 1998–2019
Source: Int J Environ Res Public Health. 2022 Mar 2;19(5):2898. doi: 10.3390/ijerph19052898 (PMC8910621; doi:10.3390/ijerph19052898)
Supplement: Supplementary file 1 [file ijerph-19-02898-s001.zip › ijerph-1580785-SI.pdf]

Supplementary Material

Evaluating Completeness of Foodborne Outbreak Reporting in the United States, 1998-2019

Yutong Zhang [1,*], Ryan B. Simpson [1], Lauren E. Sallade [1], Emily Sanchez [1], Kyle M. Monahan [2] and Elena N. Naumova [1,*]

[1] Division of Nutrition Epidemiology and Data Science, Tufts University Friedman School of Nutrition Science and Policy, 150 Harrison Avenue, Boston, MA 02111, USA; ryan.simpson@tufts.edu (R.B.S.); lauren.sallade@gmail.com (L.E.S.); emily.sanchez@tufts.edu (E.S.)

[2] Gordon Institute, Tufts University School of Engineering, 200 Boston Avenue, Medford, MA 02155, USA; kyle.monahan@tufts.edu

* Correspondence: zyutong27@gmail.com (Y.Z.); elena.naumova@tufts.edu (E.N.N.); Tel.: +1-515-817-3850 (Y.Z.); +1-617-636-2927 (E.N.N.)

Supplementary Table S1. Variable index in NORS with data cleaning procedure, where indicator represents identification variables that used to permit alignment and merging of other variables across data tables, removed indicates variables that removed from this study, no change represents variables included in the analysis without further cleaning, combined represents multiple-choice variables that combined into the count of multiple-choice options per question, conditional represents variables whose completeness depended on other relevant variables, duplicated represents variables with multiple answers for a single outbreak, where multiple answers were summed to a single answer.

Section	Data Table & Sheet	Variable Name	Format	Comments	Data Cleaning Procedure
General Information	NORSMain	CDCID	Number	CDC Report ID. Links record from a single NORS report across all tables of relational database. Multiple instances of the same CDCID within a table refer to multiple entries for one record (e.g., one outbreak with two etiologies)	Indicator
General Information	NORSMain	eFORSID	Number	This is the unique ID previously assigned to a report originally entered in eFORS. All previous eFORS reports have been migrated into NORS.	removed
General Information	NORSMain	StateID	Text	The report ID entered by the reporting site. The StateID is unique to each report created by a NORS reporting agency.	removed

General Information	NORSMain	NORSUserID	Text	User ID of person who created the report	removed
General Information	NORSMain	RecordStatus	Text	Indicates whether the report is active, finalized, or deleted	removed
General Information	NORSMain	CDCStatus	Text	NORS value for internal data management processes. Indicates whether a report has been excluded (e.g., due to multistate consolidation), cleaned, or closed out (data cleaning status applied for NORS reports for some primary modes)	removed
General Information	NORSMain	CDCWaterStatus	Text	NORS value that indicates whether CDC has classified a waterborne disease outbreak report as excluded, cleaned, or closed out as a result of routine data cleaning and review activities.	removed
General Information	NORSMain	StartTimeStamp	MM/DD/YYYY Time	Date NORS report was created.	removed
General Information	NORSMain	FinalTimeStamp	MM/DD/YYYY Time	Date NORS report was most recently finalized, if any.	removed
General Information	NORSMain	PrimaryMode	Text	Primary mode of transmission	removed
General Information	NORSMain	InvestigationComments	Text	Reporting site comments relating to the selected Investigation Method(s). See Investigation Method table/tab for selected investigation method(s)	removed
General Information	NORSMain	DateFirstIll	MM/DD/YYYY	Earliest date of reported illness onset.	no change
General Information	NORSMain	DateLastIll	MM/DD/YYYY	Latest date of reported illness onset.	no change
General Information	NORSMain	InitialExposure	MM/DD/YYYY	Earliest date of reported exposure.	no change
General Information	NORSMain	LastExposure	MM/DD/YYYY	Latest date of reported exposure.	no change
General Information	NORSMain	CDCReportDate	MM/DD/YYYY	Date of report to CDC other than the NORS report.	removed
General Information	NORSMain	LocalReportDate	MM/DD/YYYY	Date of report to local authorities.	removed

General Information	NORSMain	ExposureState	Text	The state where the exposure occurred. For a single state of exposure, the state will be listed. For multiple states of exposure, "multistate exposure" will be listed. See the States table for information on all states involved in this outbreak (i.e., state of exposure, state of residency)	no change
General Information	NORSMain	MultiStateExposure	True/False, 1/0, or -1/0	Exposure occurred in multiple states - see "OtherStates" tab/table for list of states involved. 0="False", 1 or -1="True" Format depends on export and import program	no change
General Information	NORSMain	MultiStateResidence	True/False, 1/0, or -1/0	Exposure occurred in a single state, but cases resided in another state or multiple states. 0="False", 1 or -1 ="True" Format depends on export and import program - see "States" tab/table for list of states involved.	no change
General Information	NORSMain	ExposureCounty	Text	Name of county within the exposure state where exposure occurred.	no change
General Information	NORSMain	MultiCountyExposure	True/False, 1/0, or -1/0	Exposure occurred in multiple counties - see "Counties" tab/table for list of counties involved. 0="False", 1 o r-1 ="True" Format depends on export and import program	no change
General Information	NORSMain	MultiCountyResidence	True/False, 1/0, or -1/0	Exposure occurred in a single county, but cases resided in another county or multiple counties. 0="False", 1 or -1 ="True" Format depends on export and import program - see "Counties" tab/table for list of counties involved.	no change
General Information	NORSMain	ExposureLocation	Text	Name of city, town, or place of exposure	no change
General Information	NORSMain	ConfirmedPrimary	Number	Number of laboratory confirmed primary cases	no change
General Information	NORSMain	ProbablePrimary	Number	Number of probable primary cases	no change

General Information	NORSMain	EstimatedPrimary	Number	Estimated total number of primary cases, including lab-confirmed and probable, based on the outbreak-specific definition.	no change
General Information	NORSMain	DeathsNum	Number	Number of primary cases who died	no change
General Information	NORSMain	DeathsInfo	Number	Total number of primary cases for whom information on survival is available	no change
General Information	NORSMain	HospitalNum	Number	Number of primary cases who were hospitalized	no change
General Information	NORSMain	HospitalInfo	Number	Total number of primary cases for whom information on hospitalization is available	no change
General Information	NORSMain	ERNum	Number	Number of primary cases who visited an emergency room	no change
General Information	NORSMain	ERInfo	Number	Total number of primary cases for whom information on emergency room visit is available	no change
General Information	NORSMain	HealthcareNum	Number	Number of primary cases who visited a health care provider	no change
General Information	NORSMain	HealthcareInfo	Number	Total number of primary cases for whom information on health care visit is available	no change
General Information	NORSMain	NumberMale	Number	Number of primary cases who are male	no change
General Information	NORSMain	PercentMale	Number	Percentage of primary cases who are male	removed
General Information	NORSMain	NumberFemale	Number	Number of primary cases who are female	no change
General Information	NORSMain	PercentFemale	Number	Percentage of primary cases who are female	removed
General Information	NORSMain	NumberSexUnknown	Number	Number of primary cases of unknown sex	no change
General Information	NORSMain	PercentSexUnknown	Number	Percentage of primary cases of unknown sex	removed
General Information	NORSMain	NumberAgeUnder1	Number	Number of primary cases age less than one year	combined
General Information	NORSMain	PercentAgeUnder1	Number	Percentage of primary cases aged less than one year	removed

General Information	NORSMain	NumberAge1to4	Number	Number of primary cases age one to four years	combined
General Information	NORSMain	PercentAge1to4	Number	Percentage of primary cases age one to four years	removed
General Information	NORSMain	NumberAge5to9	Number	Number of primary cases age five to nine years	combined
General Information	NORSMain	PercentAge5to9	Number	Percentage of primary cases age five to nine years	removed
General Information	NORSMain	NumberAge10to19	Number	Number of primary cases age ten to nineteen years	combined
General Information	NORSMain	PercentAge10to19	Number	Percentage of primary cases age 10 to 19 years	removed
General Information	NORSMain	NumberAge20to49	Number	Number of primary cases age 20 to 49 years	combined
General Information	NORSMain	PercentAge20to49	Number	Percentage of primary cases age 20 to 49 years	removed
General Information	NORSMain	NumberAge50to74	Number	Number of primary cases age 50 to 74 years	combined
General Information	NORSMain	PercentAge50to74	Number	Percentage of primary cases age 50 to 74 years	removed
General Information	NORSMain	NumberAge75plus	Number	Number of primary cases over 75 years of age	combined
General Information	NORSMain	PercentAge75plus	Number	Percentage of primary cases over 75 years of age	removed
General Information	NORSMain	NumberAgeUnknown	Number	Number of primary cases of unknown age	combined
General Information	NORSMain	PercentAgeUnknown	Number	Percentage of primary cases of unknown age	removed
General Information	NORSMain	IncShort	Number	Shortest incubation period, in selected units	no change
General Information	NORSMain	IncShortUnit	Text	Units of shortest incubation time	no change
General Information	NORSMain	IncMedian	Number	Median incubation period, in selected units	no change
General Information	NORSMain	IncMedianUnit	Text	Units of median incubation time	no change
General Information	NORSMain	IncLong	Number	Longest incubation period, in selected units	no change
General Information	NORSMain	IncLongUnit	Text	Units of longest incubation time	no change

General Information	NORSMain	IncubationNum	Number	Number of primary cases for whom information on incubation period is available.	no change
General Information	NORSMain	IncUnknown	True/False, 1/0, or -1/0	Information on incubation period is unknown. 0="False", 1 or -1 ="True" Format depends on export and import program	no change
General Information	NORSMain	DurShort	Number	Shortest duration time, in selected units	no change
General Information	NORSMain	DurShortUnit	Text	Units of shortest duration time	no change
General Information	NORSMain	DurMedian	Number	Median duration time, in selected units	no change
General Information	NORSMain	DurMedianUnit	Text	Units of median duration time	no change
General Information	NORSMain	DurLong	Number	Longest duration time, in selected units	no change
General Information	NORSMain	DurLongUnit	Text	Units of longest duration time	no change
General Information	NORSMain	DurationNum	Number	Number of primary cases for whom information on duration of illness is available.	no change
General Information	NORSMain	DurUnknown	True/False, 1/0, or -1/0	Information on duration of illness is unknown. 0="False", 1 or -1 ="True" Format depends on export and import program	no change
General Information	NORSMain	ConfirmedSecondary	Number	Number of laboratory confirmed secondary cases	no change
General Information	NORSMain	ProbableSecondary	Number	Number of probable secondary cases	no change
General Information	NORSMain	TotalSecondary	Number	Estimated total number of secondary cases, including lab-confirmed and probable, based on the outbreak-specific definition	no change
General Information	NORSMain	TotalCases	Number	Sum of all primary and secondary cases reported in the EstimatedPrimary and TotalSecondary fields	no change

General Information	NORSMain	NEARSID1	Text	This field is used to link NORS reports to environmental health investigation reports in the National Environmental Assessment Reporting System (NEARS) (formerly Environmental Health Specialists Network)	removed
General Information	NORSMain	NEARSID2	Text	This field is used to link NORS reports to environmental health investigation reports in the National Environmental Assessment Reporting System (NEARS) (formerly Environmental Health Specialists Network)	removed
General Information	NORSMain	NEARSID3	Text	This field is used to link NORS reports to environmental health investigation reports in the National Environmental Assessment Reporting System (NEARS) (formerly Environmental Health Specialists Network)	removed
General Information	NORSMain	NEARSID4	Text	This field is used to link NORS reports to environmental health investigation reports in the National Environmental Assessment Reporting System (NEARS) (formerly Environmental Health Specialists Network)	removed
General Information	NORSMain	OHHABSID1	Text	This field is used to link NORS reports to reports in the One Health Harmful Algal Bloom System (OHHABS)	removed
General Information	NORSMain	OHHABSID2	Text	This field is used to link NORS reports to reports in the One Health Harmful Algal Bloom System (OHHABS)	removed
General Information	NORSMain	TracebackConducted	True/False, 1/0, or -1/0	Traceback was conducted. 0="False", 1 or -1 ="True". See Traceback tab/table for full traceback	no change

				information. Format depends on export and import program	
General Information	NORSMain	Recall	True/False, 1/0, or -1/0	A food or bottled water product was recalled. 0="False", 1 or -1="True". Format depends on export and import program	no change
General Information	NORSMain	RecallItem	Text	Type of item recalled	no change
General Information	NORSMain	RecallComments	Text	Comments about the recall.	removed
General Information	NORSMain	ReportingSite	Text	Reporting site of the report author	removed
General Information	NORSMain	AgencyState	Text	State of report agency	removed
General Information	NORSMain	AgencyName	Text	Name of the NORS agency reporting the outbreak	removed
General Information	NORSMain	AgencyContact	Text	Name of primary agency contact	removed
General Information	NORSMain	AgencyTitle	Text	Title of agency contact	removed
General Information	NORSMain	AgencyEmail	Text	Email address of agency contact	removed
General Information	NORSMain	AgencyPhone	Text	Phone number of agency contact	removed
General Information	NORSMain	AgencyFax	Text	Fax number of agency contact	removed
General Information	NORSMain	GeneralComments	Text	General comments about the outbreak	removed
General Information	NORSMain	CDCRemarks	Text	General comments added by CDC NORS managing administrators	removed
General Information	NORSMain	References	Text	Publications or other references related to outbreak added by CDC	removed
General Information	NORSMain	Attachment	True/False, 1/0, or -1/0	Indicates whether or not one or more attachments have been included in the NORS report. 0="False", 1 or -1="True" Format depends on export and import program	removed

General Information	InvestigationMethod	CDCID	Number	CDC Report ID. Links record from a single NORS report across all tables of relational database. Multiple instances of the same CDCID within a table refer to multiple entries for one record (e.g., one outbreak with two etiologies)	Indicator
General Information	InvestigationMethod	InvestigationMethod	Text	Name of investigation method used to investigate outbreak	duplicate removed
General Information	Symptoms	CDCID	Number	CDC Report ID. Links record from a single NORS report across all tables of relational database. Multiple instances of the same CDCID within a table refer to multiple entries for one record (e.g., one outbreak with two etiologies)	Indicator
General Information	Symptoms	Symptom	Text	Name of sign or symptom	duplicate removed
General Information	Symptoms	SymptomCases	Number	Number of primary cases with sign or symptom	conditional
General Information	Symptoms	SymptomInfo	Number	Total number of primary cases for whom information on specified sign or symptom is available	conditional
General Information	SecondaryTransmissionMode	CDCID	Number	CDC Report ID. Links record from a single NORS report across all tables of relational database. Multiple instances of the same CDCID within a table refer to multiple entries for one record (e.g., one outbreak with two etiologies)	Indicator
General Information	SecondaryTransmissionMode	SecondaryMode	Text	Confirmed or suspected mode of secondary transmission	duplicate removed
General Information	EforsAge	CDCID	Number	CDC Report ID. Links record from a single NORS report across all tables of relational database. Multiple instances of the same CDCID within a table refer to multiple entries for one record (e.g., one outbreak with two etiologies)	Indicator

General Information	EforsAge	AgeLessThan1	Number	Approximate percentage of cases in each age group among the total number of primary cases for whom information is available	combined
General Information	EforsAge	Age1to4	Number	Approximate percentage of cases in each age group among the total number of primary cases for whom information is available	combined
General Information	EforsAge	Age5to19	Number	Approximate percentage of cases in each age group among the total number of primary cases for whom information is available	combined
General Information	EforsAge	Age20to49	Number	Approximate percentage of cases in each age group among the total number of primary cases for whom information is available	combined
General Information	EforsAge	AgeGreaterThanEqual50	Number	Approximate percentage of cases in each age group among the total number of primary cases for whom information is available	combined
General Information	EforsAge	AgeUnknown	Number	Approximate percentage of cases in each age group among the total number of primary cases for whom information is available	combined
Food Information	EforsFoodContributingFactor	CDCID	Number	CDC Report ID. Links record from a single NORS report across all tables of relational database. Multiple instances of the same CDCID within a table refer to multiple entries for one record (e.g., one outbreak with two etiologies)	Indicator
Food Information	EforsFoodContributingFactor	ContributingFactorCode	Text	Code of contributing factor	combined
Food Information	EforsFoodContributingFactor	ContributingFactorDescription	Text	Description of contributing factor	combined
Food Information	EforsFoodContributingFactor	ContributingFactorTypeName	Text	contributing factor type name	Removed

Food Information	FB_FoodMain	CDCID	Number	CDC Report ID. Links record from a single NORS report across all tables of relational database. Multiple instances of the same CDCID within a table refer to multiple entries for one record (e.g., one outbreak with two etiologies)	Indicator
Food Information	FB_FoodMain	FoodVehicleUndetermined	True/False, 1/0, -1/0	Indicates if a food vehicle was not identified for the outbreak. Format depends on export and import program, 0="False", 1 or -1 ="True".	no change
Food Information	FB_FoodMain	ReasonFoodEpi	True/False, 1/0, -1/0	Indicates whether epidemiologic evidence determined that the outbreak was foodborne, with an undetermined vehicle.	combined
Food Information	FB_FoodMain	ReasonFoodLab	True/False, 1/0, -1/0	Indicates whether laboratory evidence determined that the outbreak was foodborne, with an undetermined vehicle.	combined
Food Information	FB_FoodMain	ReasonFoodTrace	True/False, 1/0, -1/0	Indicates whether traceback or environmental evidence determined that the outbreak was foodborne, with an undetermined vehicle.	combined
Food Information	FB_FoodMain	ReasonFoodOther	True/False, 1/0, -1/0	Indicates whether other evidence determined that the outbreak was foodborne, with an undetermined vehicle.	combined
Food Information	FB_FoodMain	KitchenManager	0/1/7/Null (blank)	Indicates whether a kitchen manager at the location of preparation was certified in food safety (e.g., ServSafe). 0="No", 1="True", 7="Unknown", null (blank) = no response	conditional
Food Information	FB_FoodMain	ContributingFactorsUnknown	True/False, 1/0, -1/0	Indicates if contributing factor is unknown, 0="False", 1 or -1="True". Format depends on export and import program	no change
Food Information	FB_FoodMain	FoodWorkerImplicated	0/1/7/Null (blank)	Indicates if food worker was implicated, 0="No", 1="True", 7="Unknown", null (blank) = no response	combined

Food Information	FB_FoodMain	FoodWorkerImplicatedName	Text	If food worker was implicated, indicates type of evidence that implicated the food worker	combined
Food Information	FB_FoodMain	ArchivedTotalExpFood	Number	NORS question collected between Jan 2009 and May 2011. This question is no longer being collected, but the data entered during this period has been archived for reference. This variable indicates the number of cases exposed to at least one of the implicated foods reported	removed
Food Information	FB_FoodborneContaminationPoint	CDCID	Number	CDC Report ID. Links record from a single NORS report across all tables of relational database. Multiple instances of the same CDCID within a table refer to multiple entries for one record (e.g., one outbreak with two etiologies)	Indicator
Food Information	FB_FoodborneContaminationPoint	ContaminationPoint	Text	Indicates if point of contamination occurred "Before preparation," "Preparation," or "Unknown."	no change
Food Information	FB_FoodborneContaminationPoint	BeforePrepPreHarvest	True/False, 1/0, -1/0	If point of contamination occurred 'before preparation', variable indicates during preharvest, 0="False", 1 or -1 ="True". Format depends on export and import program	removed
Food Information	FB_FoodborneContaminationPoint	BeforePrepProcessing	True/False, 1/0, -1/0	If point of contamination occurred 'before preparation', variable indicates during processing, 0="False", 1 or -1="True". Format depends on export and import program	removed
Food Information	FB_FoodborneContaminationPoint	BeforePrepUnknown	True/False, 1/0, -1/0	If point of contamination occurred 'before preparation', variable indicates when is unknown, 0="False", 1 or -1="True". Format depends on export and import program	removed

Food Information	FB_FoodborneContaminationPoint	EnvironmentalEvidence	True/False, 1/0, -1/0	Environmental evidence is the reason why the confirmed or suspected point of contamination was assumed, 0="False", 1 or -1="True". Format depends on export and import program	combined
Food Information	FB_FoodborneContaminationPoint	EpidemiologicEvidence	True/False, 1/0, -1/0	Indicates if epidemiologic evidence is the reason why the confirmed or suspected point of contamination was assumed, 0="False", 1 or -1="True". Format depends on export and import program	combined
Food Information	FB_FoodborneContaminationPoint	LabEvidence	True/False, 1/0, -1/0	Lab evidence is the reason why the confirmed or suspected point of contamination was assumed, 0="False", 1 or -1="True". Format depends on export and import program	combined
Food Information	FB_FoodborneContaminationPoint	PriorExperience	True/False, 1/0, -1/0	Indicates if prior experience is the reason why the confirmed or suspected point of contamination was assumed, 0="False", 1 or -1="True". Format depends on export and import program	combined
Food Information	FB_FoodImplicated	CDCID	Number	CDC Report ID. Links record from a single NORS report across all tables of relational database. Multiple instances of the same CDCID within a table refer to multiple entries for one record (e.g., one outbreak with two etiologies)	Indicator
Food Information	FB_FoodImplicated	FoodImplicatedID	Number	NORS value for internal data management processes. Identifies the individual implicated food during the outbreak; each outbreak may have multiple implicated foods. Variable is a primary key.	
Food Information	FB_FoodImplicated	FoodName	Text	Implicated food(s)	duplicate removed
Food Information	FB_FoodImplicated	FoodConfirmed	Text	Indicates whether the vehicle was confirmed or suspected.	combined

Food Information	FB_FoodImplicated	FoodExposed	Number	Number of cases exposed to the implicated food	combined
Food Information	FB_FoodImplicated	Imported	0/1/7/Null (blank)	Indicates if the implicated food was imported into the US, 0 ="No", 1 or -1= "Yes" 7= "Unknown", Null (blank)=No Response	no change
Food Information	FB_FoodImplicated	CountryName	Text	If the contaminated food was imported, indicate the name of the country if known.	removed
Food Information	FB_FoodImplicated	ProduceAndSold	0/1/7/Null (blank)	Indicates if the food product was both produced under domestic regulatory oversight (commercial product produced within US that is regulated by the FDA) and sold, 0 ="No", 1= "Yes", 7= "Unknown", Null (blank)=No Response	no change
Food Information	FB_ReasonSuspected	FoodImplicatedID	Number	NORS value for internal data management processes. Identifies the individual implicated food during the outbreak; each outbreak may have multiple implicated foods. Variable is a primary key.	Indicator
Food Information	FB_ReasonSuspected	ReasonSuspected	Text	For the implicated food, indicates reason(s) suspected or confirmed.	Conditional
Food Information	FB_Ingredients	FoodImplicatedID	Number	NORS value for internal data management processes. Identifies the individual implicated food during the outbreak; each outbreak may have multiple implicated foods. Variable is a primary key.	Indicator
Food Information	FB_Ingredients	IngredientName	Text	For the implicated food suspected or investigated, indicates a single ingredient	combined
Food Information	FB_Ingredients	ContaminatedIngredient	True/False, 1/0, or -1/0	Among the ingredients previously listed, indicates a contaminated ingredient(s), 0="False", 1 or -1 ="True" Format depends on export and import program	combined
Food Information	FB_MethodofProc	FoodImplicatedID	Number	NORS value for internal data management processes. Identifies the individual implicated food	Indicator

				during the outbreak; each outbreak may have multiple implicated foods. Variable is a primary key.	
Food Information	FB_MethodofProc	MethodofProcessing	Text	For the implicated food, indicates method of processing	conditional
Food Information	FB_MethodofPrep	FoodImplicatedID	Number	NORS value for internal data management processes. Identifies the individual implicated food during the outbreak; each outbreak may have multiple implicated foods. Variable is a primary key.	Indicator
Food Information	FB_MethodofPrep	MethodofPreparation	Text	For the implicated food, indicates method of preparation.	conditional
Food Information	EforsFoodMethodOfPreparation	CDCID	Number	CDC Report ID. Links record from a single NORS report across all tables of relational database. Multiple instances of the same CDCID within a table refer to multiple entries for one record (e.g., one outbreak with two etiologies)	Indicator
Food Information	EforsFoodMethodOfPreparation	FoodImplicatedID	Number	NORS value for internal data management processes. Identifies the individual implicated food during the outbreak; each outbreak may have multiple implicated foods. Variable is a primary key.	Indicator
Food Information	EforsFoodMethodOfPreparation	MethodOfPreparationName	Text	For the implicated food, indicates method of preparation.	conditional
Food Information	EforsFoodMethodOfPreparation	CodeOfPreparation	Text	For the implicated food, indicates method of preparation code	Removed
Food Information	FB_LevelofPrep	FoodImplicatedID	Number	NORS value for internal data management processes. Identifies the individual implicated food during the outbreak; each outbreak may have multiple implicated foods. Variable is a primary key.	Indicator
Food Information	FB_LevelofPrep	LevelofPreparation	Text	For the implicated food, indicates level of preparation	conditional

Food Information	FB_WherePrep	CDCID	Number	CDC Report ID. Links record from a single NORS report across all tables of relational database. Multiple instances of the same CDCID within a table refer to multiple entries for one record (e.g., one outbreak with two etiologies)	Indicator
Food Information	FB_WherePrep	WherePrepName	Text	Location of exposure of where food was prepared	duplicate removed
Food Information	FB_WhereEaten	CDCID	Number	CDC Report ID. Links record from a single NORS report across all tables of relational database. Multiple instances of the same CDCID within a table refer to multiple entries for one record (e.g., one outbreak with two etiologies)	Indicator
Food Information	FB_WhereEaten	WhereEatenName	Text	Location of exposure of where food was eaten	duplicate removed
Food Information	FB_ContributingFactors	CDCID	Number	CDC Report ID. Links record from a single NORS report across all tables of relational database. Multiple instances of the same CDCID within a table refer to multiple entries for one record (e.g., one outbreak with two etiologies)	Indicator
Food Information	FB_ContributingFactors	ContributingFactorName	Text	Name of contributing factor	combined
Food Information	FB_ContributingFactors	ContributingFactorCode	Text	Code of contributing factor	combined
Food Information	FB_ContributingFactors	ContributingFactorType	Text	Type of contributing factor	combined
Food Information	FB_School	CDCID	Number	CDC Report ID. Links record from a single NORS report across all tables of relational database. Multiple instances of the same CDCID within a table refer to	Indicator

				multiple entries for one record (e.g., one outbreak with two etiologies)	
Food Information	FB_School	MultipleSchools	True/False, 1/0, or -1/0	Did the outbreak involve a single or multiple schools? Format depends on export and import program	combined
Food Information	FB_School	NumOfMultipleSchools	Number	Number of schools if multiple schools were involved in the outbreak	combined
Food Information	FB_School	TotalEnrollment	Number	Approximate number of students enrolled in the school	combined
Food Information	FB_School	UnknownEnrollmentNumber	True/False, 1/0, or -1/0	Indicates if the number of students enrolled is unknown. Format depends on import and export program, 0="False", 1 or -1="True".	combined
Food Information	FB_School	SchoolFundingName	Text	Primary funding of involved schools	no change
Food Information	FB_School	SchoolInspectedName	Text	Indicates how many times the school cafeteria or kitchen has been inspected in the last 12 months by state, county, or local health departments.	no change
Food Information	FB_School	HACCP	0/1/7/Null (blank)	Indicate whether the school involved in the outbreak has a HACCP plan in place for the school feeding program, 0 ="No", 1= "Yes", 7= "Unknown or Undetermined", Null (blank)= No Response	no change
Food Information	FB_School	NationalSchoolProgram	0/1/7/Null (blank)	Was implicated food item provided to the school through the National School Lunch/Breakfast Program? 0 ="No", 1= "Yes", 7= "Unknown or Undetermined", Null (blank)= No Response	no change
Food Information	FB_School	FoodItemPurchasedDonatedBy	Text	If school participated in the National School Lunch/Breakfast program, indicates the source of the implicated food items	removed

Food Information	FB_SchoolLevel	CDCID	Number	CDC Report ID. Links record from a single NORS report across all tables of relational database. Multiple instances of the same CDCID within a table refer to multiple entries for one record (e.g., one outbreak with two etiologies)	Indicator
Food Information	FB_SchoolLevel	GradeLevel	Text	Indicates grade level of the students in the outbreak	duplicate removed
Food Information	FB_SchoolPrep	CDCID	Number	CDC Report ID. Links record from a single NORS report across all tables of relational database. Multiple instances of the same CDCID within a table refer to multiple entries for one record (e.g., one outbreak with two etiologies)	Indicator
Food Information	FB_SchoolPrep	SchoolFoodPrepName	Text	Indicates the preperation of the implicated item	duplicate removed
Food Information	FB_GroundBeef	CDCID	Number	CDC Report ID. Links record from a single NORS report across all tables of relational database. Multiple instances of the same CDCID within a table refer to multiple entries for one record (e.g., one outbreak with two etiologies)	Indicator
Food Information	FB_GroundBeef	PercentageGBConsumed	Number	Percentage of ill persons that ate ground beef raw or undercooked	no change
Food Information	FB_GroundBeef	CaseReady	0/1/7/Null (blank)	Indicates whether the ground beef was case ready, 0="No", 1= "Yes", 7= "Unknown", Null (blank)= No Response	no change
Food Information	FB_GroundBeef	GBReground	0/1/7/Null (blank)	Indicates if the beef was ground or reground by the retailer, 0="No", 1= "Yes", 7= "Unknown", Null (blank)= No Response	no change
Food Information	FB_Egg	CDCID	Number	CDC Report ID. Links record from a single NORS report across all tables of relational database. Multiple instances of the same CDCID within a table refer to multiple entries for one record (e.g., one outbreak with two etiologies)	Indicator

Food Information	FB_Egg	EggName	Text	Indicate if the eggs were 'in shell, unpasteurized,' 'in shell, pasteurized,' 'packaged liquid or dry,' 'stored with inadequate refrigeration during or after sale,' 'consumed raw,' 'consumed undercooked,' or 'pooled'	no change
Food Information	FB_Egg	SEFoundOnFarm	0/1/7/Null (blank)	Indicates if Salmonella enteritis was identified at the farm where the eggs originated, 0= "No", 1= "Yes", 7= "Unknown", Null (blank)= No Response	no change
Etiology Information	GenLab	CDCID	Number	CDC Report ID. Links record from a single NORS report across all tables of relational database. Multiple instances of the same CDCID within a table refer to multiple entries for one record (e.g., one outbreak with two etiologies)	Indicator
Etiology Information	GenLab	SpecimenCollected	0/1/7/Null (blank)	Indicates whether any specimens or environmental, food, or water samples were collected and tested. 0="False", 1 or -1 ="True" Format depends on export and import program	no change
Etiology Information	GenLab	SampleTypeHuman	0/1/7/Null (blank)	Indicates whether human samples were tested. 0="False", 1 or -1 ="True" Format depends on export and import program	combined
Etiology Information	GenLab	SampleTypeHumanNum	Number	Number of human specimens tested	combined
Etiology Information	GenLab	SampleTypeAnimal	0/1/7/Null (blank)	Indicates whether animal samples were tested. 0="False", 1 or -1 ="True" Format depends on export and import program	combined
Etiology Information	GenLab	SampleTypeAnimalNum	Number	Number of animal specimens tested	combined
Etiology Information	GenLab	SampleTypeFood	0/1/7/Null (blank)	Indicates whether food samples were tested. 0="False", 1 or -1	combined

				="True" Format depends on export and import program	
Etiology Information	GenLab	SampleTypeFood Num	Number	Number of food specimens tested	combined
Etiology Information	GenLab	SampleTypeWater	0/1/7/Null (blank)	Indicates whether water samples were tested. 0="False", 1 or -1 ="True" Format depends on export and import program	combined
Etiology Information	GenLab	SampleTypeWater Num	Number	Number of water specimens tested	combined
Etiology Information	GenLab	SampleTypeOther	0/1/7/Null (blank)	Indicates whether other samples were tested, specify in general remarks section. 0="False", 1 or -1 ="True" Format depends on export and import program	combined
Etiology Information	GenLab	SampleTypeOther Num	Number	Number of other enviornmental specimens tested	combined
Etiology Information	GenLab	TestedBacteria	True/False, 1/0, or -1/0	This field indicates whether collected specimens, if any, were tested for bacterial etiologies. 0="False", 1 or -1="True" Format depends on export and import program	combined
Etiology Information	GenLab	TestedViruses	True/False, 1/0, or -1/0	This field indicates whether collected specimens, if any, were tested for viral etiologies. 0="False", 1 or -1="True" Format depends on export and import program	combined
Etiology Information	GenLab	TestedParasites	True/False, 1/0, or -1/0	This field indicates whether collected specimens, if any, were tested for parasitic etiologies. 0="False", 1or -1="True" Format depends on export and import program	combined
Etiology Information	GenLab	TestedChemicals	True/False, 1/0, or -1/0	This field indicates whether collected specimens, if any, were tested for chemical etiologies. 0="False", 1 or -1 ="True" Format depends on export and import program	combined

Etiology Information	GenLab	TestedUnknown	True/False, 1/0, or -1/0	This field indicates that collected specimens/samples were tested, but for what they were tested is unknown. 0="False", 1or -1="True" Format depends on export and import program	combined
Etiology Information	GenLab	TestTypeChemical	True/False, 1/0, or -1/0	Indicates that specimens/samples were tested using chemical testing methods. 0="False", 1 or -1="True" Format depends on export and import program	combined
Etiology Information	GenLab	TestTypeCulture	True/False, 1/0, or -1/0	Indicates that specimens/samples were tested using culture methods. 0="False", 1or -1="True" Format depends on export and import program	combined
Etiology Information	GenLab	TestTypePCR	True/False, 1/0, or -1/0	Indicates that specimens/samples were tested using DNA or RNA amplification methods. 0="False", 1or -1="True" Format depends on export and import program	combined
Etiology Information	GenLab	TestTypeMicro	True/False, 1/0, or -1/0	Indicates that specimens/samples were tested using microscopy methods. 0="False", 1or -1="True" Format depends on export and import program	combined
Etiology Information	GenLab	TestTypeSero	True/False, 1/0, or -1/0	Indicates that specimens/samples were tested using serological or imuunological methods. 0="False", 1or -1="True" Format depends on export and import program	combined
Etiology Information	GenLab	TestTypeTissue	True/False, 1/0, or -1/0	Indicates that specimens/samples were tested using tissue culture infectivity assay. 0="False", 1or -1="True" Format depends on export and import program	combined
Etiology Information	GenLab	TestTypeOther	True/False, 1/0, or -1/0	Indicates that specimens/samples were tested using another method. 0="False", 1or -1="True" Format	combined

				depends on export and import program	
Etiology Information	GenLab	TestTypeUnknown	True/False, 1/0, or -1/0	Indicates that specimens/samples were tested using an unknown methods. 0="False", 1or -1="True" Format depends on export and import program	combined
Etiology Information	GenLab	ASTPerformed	0/1/7/Null (blank)	Indicates whether any samples were tested for antimicrobial resistance. 0="No", 1="Yes", 7="Unknown", null (blank) = no response	no change
Etiology Information	GenLab	ASTWhereClinical	True/False, 1/0, or -1/0	Indicates if AST was done by a clinical lab (e.g., hospital). 0="False", 1or -1="True" Format depends on export and import program	removed
Etiology Information	GenLab	ASTWherePHLab	True/False, 1/0, or -1/0	Indicates if AST was done by a public health lab. 0="False", 1or -1="True" Format depends on export and import program	removed
Etiology Information	GenLab	ASTWhereNARMS	True/False, 1/0, or -1/0	Indicates if AST was done by the National Antimicrobial Resistance Monitoring System (NARMS). 0="False", 1or -1="True" Format depends on export and import program	removed
Etiology Information	GenLab	ASTWhereOther	True/False, 1/0, or -1/0	Indicates if AST was done by another lab. 0="False", 1or -1="True" Format depends on export and import program	removed
Etiology Information	GenLab	ASTWhereUnknown	True/False, 1/0, or -1/0	Indicates if AST testing is unknown. 0="False", 1or -1="True" Format depends on export and import program	removed
Etiology Information	GenLab	ASTOutbreak	0/1/7/Null (blank)	Indicates whether any antimicrobial resistant strains were associated with the outbreak. 0="False", 1="True", 7="Unknown", null (blank) = no response	removed

Etiology Information	GenLab	EtiologyKnown	True/False, 1/0, or -1/0	Indicates that there is at least one confirmed or suspected outbreak etiology (based on either laboratory confirmation or epidemiological evidence). 0="False", 1 or -1 ="True", Format depends on export and import program.	no change
Etiology Information	GenLab	ArchivedSpecimensTaken	0/1/7/Null (blank)	NORS question collected between Jan 2009 and Sept 2015. This question is no longer being collected, but the data entered during this period has been archived for reference. If etiology is unknown, were patient specimens collected? 0="False", 1="True", 7="Unknown", null (blank) = no response	removed
Etiology Information	GenLab	ArchivedNumberSpecimens	Number	NORS question collected between Jan 2009 and Sept 2015. This question is no longer being collected, but the data entered during this period has been archived for reference. Number of specimens collected for outbreak of unknown etiology.	removed
Etiology Information	GenEtiology	CDCID	Number	CDC Report ID. Links record from a single NORS report across all tables of relational database. Multiple instances of the same CDCID within a table refer to multiple entries for one record (e.g., one outbreak with two etiologies)	Indicator
Etiology Information	GenEtiology	GenusName	Text	Genus of idendified etiology	duplicate removed
Etiology Information	GenEtiology	SpeciesName	Text	Species of identified etiology	conditional
Etiology Information	GenEtiology	SerotypeName	Text	Serotype of identified etiology	conditional
Etiology Information	GenEtiology	Polymerase	Text	Polymerase type of a norovirus strain identified as an outbreak etiology	removed

Etiology Information	GenEtiology	Capsid	Text	Capsid type of a norovirus strain identified as an outbreak etiology	removed
Etiology Information	GenEtiology	OtherCharacteristics	Text	Other characteristics of identified etiology	removed
Etiology Information	GenEtiology	Confirmed	Text	Indicates whether or not the listed etiology is laboratory 'Confirmed' or 'Suspected'.	removed
Etiology Information	GenEtiology	NumberLabConfirmed	Number	Number of lab confirmed cases	removed
Etiology Information	GenEtiology	EtiologyBacteria	True/False, 1/0, or -1/0	The listed etiology is bacterial. Value is automatically assigned for analysis purposes. 0="False", 1 or -1="True" Format depends on export and import program	removed
Etiology Information	GenEtiology	EtiologyChemicals	True/False, 1/0, or -1/0	The listed etiology is chemical. Value is automatically assigned for analysis purposes. 0="False", 1 or -1="True" Format depends on export and import program	removed
Etiology Information	GenEtiology	EtiologyViruses	True/False, 1/0, or -1/0	The listed etiology is viral. Value is automatically assigned for analysis purposes. 0="False", 1 or -1 ="True" Format depends on export and import program	removed
Etiology Information	GenEtiology	EtiologyParasites	True/False, 1/0, or -1/0	The listed etiology is parasitic. Value is automatically assigned for analysis purposes. 0="False", 1="True" Format depends on export and import program	removed
Etiology Information	GenEtiology	EtiologyHepatitis	True/False, 1/0, or -1/0	The listed etiology is hepatitis. Value is automatically assigned for analysis purposes. 0="False", 1="True" Format depends on export and import program	removed
Etiology Information	GenEtiology	EtiologySourceSystem	Text	Etiology source system name	removed

Supplementary Table S2. Crude variable completeness estimates across all outbreaks and pathogens, listed by categories, Category 1 (100-95% completeness; 18 variables), Category 2 (94-70% completeness; 21 variables), Category 3 (69-35% completeness; 22 variables), Category 4 (34-25% completeness; 20 variables), and Category 5 (24-0% completeness, 22 variables).

Category	Variable Name	Comments	Crude Completeness
Category 1	Exposure State	The state where the exposure occurred.	100.00%
	MultiStateExposure	Exposure occurred in multiple states	100.00%
	MultiStateResidence	Exposure occurred in a single state, but cases resided in another state or multiple states.	100.00%
	EstimatedPrimary	Estimated number of primary cases	100.00%
	TotalCases	Sum of all primary and secondary cases reported in the EstimatedPrimary and TotalSecondary fields	100.00%
	ExposureStateTotal	Indicates states(s) where exposure occurred in a single or multistate exposure outbreak.	100.00%
	ResidenceStateTotal	Number of states where persons were exposed in multistate outbreak	100.00%
	EtiologyKnown	Indicates that there is at least one confirmed or suspected outbreak etiology (based on either laboratory confirmation or epidemiological evidence).	99.42%
	FoodVehicleUndetermined	Indicates if a food vehicle was not identified for the outbreak.	99.38%
	Contributing FactorsUnknown	Indicates if contributing factor is unknown	99.38%
	SymptomInfo	Total number of primary cases for whom information on specified sign or symptom is available	98.99%
	MultiCountyResidence	Exposure occurred in a single county, but cases resided in another county or multiple counties.	97.69%
	MultiCountyExposure	Exposure occurred in multiple counties	97.48%
	SymptomCases	Number of primary cases with sign or symptom	97.32%
	WhereEatenName	Location of exposure of where food was eaten	95.89%
	InvestigationMethod	Name of investigation method used to investigate outbreak	95.87%
	WherePrepName	Location of exposure of where food was prepared	95.78%
	SymptomCounts	The number of sign or symptom per outbreak	95.11%
Category 2	FoodWorker ImplicatedName	If food worker was implicated, indicates type of evidence that implicated the food worker	89.92%
	foodVehicle_reason	Indicates food vehicles identified for the outbreak.	89.13%
	IncUnknown	Information on incubation period is unknown.	88.67%
	HospitalInfo	Total number of primary cases for whom information on hospitalization is available	88.47%
	hospitalNum	Number of primary cases who were hospitalized	88.06%
	ProbablePrimary	Number of probable primary cases	87.55%
	DeathsInfo	Total number of primary cases for whom information on survival is available	86.96%
	DeathsNum	Number of primary cases who died	86.78%
	IncShortUnit	Units of shortest incubation time	82.58%
	IncLongUnit	Units of longest incubation time	81.00%

	IncMedianUnit	Units of median incubation time	80.50%
	ConfirmedPrimary	Number of laboratory confirmed primary cases	80.17%
	HealthcareInfo	Total number of primary cases for whom information on health care visit is available	79.92%
	InitialExposure	Earliest date of reported exposure.	77.70%
	HealthcareNum	Number of primary cases who visited a health care provider	77.30%
	FoodName	Implicated food(s)	75.94%
	DurShortUnit	Units of shortest duration time	75.88%
	DurLongUnit	Units of longest duration time	75.42%
	LastExposure	Latest date of reported exposure.	71.59%
	DurMedianUnit	Units of median duration time	71.42%
	IncShort	Shortest incubation period, in selected units	70.45%
**Category 3**	IncLong	Longest incubation period, in selected units	68.61%
	TracebackConducted	Traceback was conducted.	67.38%
	recall	A food or bottled water product was recalled.	67.27%
	suspect_count	The number of suspected or confirmed reasons for the implicated food	66.59%
	recall_food	Type of item recalled	66.16%
	LevelOfPreparation	For the implicated food, indicates level of preparation.	66.00%
	IncMedian	Median incubation period, in selected units	64.25%
	DateLastIll	Latest date of reported illness onset.	60.29%
	DurShort	Shortest duration time, in selected units	56.31%
	DurLong	Longest duration time, in selected units	56.01%
	ExposureCounty	Name of county within the exposure state where exposure occurred.	50.54%
	age_average	The ratio of complete primary age information per outbreak	49.39%
	DurMedian	Median duration time, in selected units	48.48%
	SpeciesName	Species of identified etiology	46.72%
	Contamination	Indicates contamination factors	44.91%
	DurUnknown	Information on duration of illness is unknown	43.85%
	school_numb	The Number of schools were involved in the outbreak	42.22%
	Ingre_count	The number of ingredients invovled in the outbreak	39.79%
	ERInfo	Total number of primary cases for whom information on emergency room visit is available	38.96%
	ERNum	Number of primary cases who visited an emergency room	38.72%
	ExposureLocation	Name of city, town, or place of exposure	36.18%
	SchoolFoodPrepName	Indicates the preperation of the implicated item	35.16%
**Category 4**	NationalSchoolProgram	Was implicated food item provided to the school through the National School Lunch/Breakfast Program?	32.65%
	FoodItem PurchasedDonatedBy	If school participated in the National School Lunch/Breakfast program, indicates the source of the implicated food items	32.57%
	GradeLevel	Indicates grade level of the students in the outbreak	31.79%
	HACCP	Indicate whether the school involved in the outbreak has a HACCP plan in place for the school feeding program	31.59%
	SchoolInspectedName	Indicates how many times the school cafeteria or kitchen has been inspected in the last 12 months by state, county, or local health departments.	31.25%

	Proliferation	Indicates proliferation/amplification factors	30.58%
	TotalEnrollment	Approximate number of students enrolled in the school	30.50%
	ContaminationPoint	Indicates if point of contamination occurred "Before preparation," "Preparation," or "Unknown."	30.42%
	Survival	Indicates survival factors	30.13%
	NumberMale	Number of primary cases who are male	29.75%
	Imported	Indicates if the implicated food was imported into the US	29.54%
	NumberFemale	Number of primary cases who are female	28.51%
	Produced And Sold	Indicates if the food product was both produced under domestic regulatory oversight (commercial product produced within US that is regulated by the FDA) and sold	27.81%
	Method of Processing	For the implicated food, indicates method of processing	27.44%
	Method of Preparation	For the implicated food, indicates method of preparation.	27.06%
	IncubationNum	Number of primary cases for whom information on incubation period is available.	26.56%
	Suspect reasons	Indicates the reasons why the confirmed or suspected point of contamination was assumed, including environmental evidence, epidemiology evidence, lab evidence, prior experience.	26.15%
	KitchenManager	Indicates whether a kitchen manager at the location of preparation was certified in food safety (e.g., ServSafe).	25.93%
	Confirmed Secondary	Number of laboratory confirmed secondary cases	25.09%
	ProbableSecondary	Number of probable secondary cases	24.53%
**Category 5**	SchoolFundingName	Primary funding of involved schools	23.79%
	EggName	Indicate if the eggs were 'in shell, unpasteurized,' 'in shell, pasteurized,' 'packaged liquid or dry,' 'stored with inadequate refrigeration during or after sale,' 'consumed raw,' 'consumed undercooked,' or 'pooled'	23.16%
	DurationNum	Number of primary cases for whom information on duration of illness is available.	22.03%
	FoodExposed	Number of cases exposed to the implicated food.	21.15%
	TotalSecondary	Estimated total number of secondary cases, including lab-confirmed and probable, based on the outbreak-specific definition.	20.88%
	SpecimenCollected	Indicates whether any specimens or environmental, food, or water samples were collected and tested.	19.88%
	TestType	Indicates pecimens/samples testing methods, including chemical, culture, PCR, microscopy, serological,tissue, other, unknown.	18.71%
	Number SexUnknown	Number of primary cases of unknown sex	18.46%
	SEFoundOnFarm	Indicates if Salmonella enteritis was identified at the farm where the eggs originated.	17.82%
	test_type	Indicates whether human/animal/food/water/other samples were tested.	15.00%
	tests_human	Number of human specimens tested	13.85%
	GBReground	Indicates if the beef was ground or reground by the retailer.	13.55%
	CaseReady	Indicates whether the ground beef was case ready.	13.43%
	ASTPerformed	Indicates whether any samples were tested for antimicrobial resistance.	11.13%

	FoodConfirmed	Indicates whether the vehicle was confirmed or suspected.	10.53%
	Percentage GBConsumed	Percentage of ill persons that ate ground beef raw or undercooked	9.74%
	tests_food	Number of food specimens tested	9.42%
	tests_other	Number of other specimens tested	7.41%
	tests_animal	Number of animal specimens tested	7.26%
	tests_water	Number of water specimens tested	7.24%
	SerotypeName	Serotype of identified etiology	6.98%
	SecondaryMode	Confirmed or suspected mode of secondary transmission	3.05%

Supplementary Table S3. Summary of seasonality and % completeness change in eFORS and NORS system by categories and pathogen groups.

Category	Estimated % Completeness at the point of system changing		% Completeness change in eFORS time		% Completeness change in NORS time		Seasonality	
	estimate	*std.error*	*estimate*	*std.error*	*estimate*	*std.error*	*eFORS*	*NORS*
All Pathogens								
1	97.699**	0.14	-0.001	0.002	0.001	0.002	-	-
2	86.661**	0.428	0.167**	0.006	0.035**	0.006	-	-
3	64.081**	0.49	0.321**	0.007	0.078**	0.007	-	-
4	24.172**	0.686	0.207**	0.01	0.328**	0.01	-	-
5	4.889**	0.435	0.033**	0.006	0.396**	0.006	-	-
Norovirus								
1	98.856**	0.19	0.007*	0.003	-0.012**	0.003	-	+
2	88.915**	0.756	0.159**	0.011	0.021	0.011	-	-
3	66.305**	0.737	0.335**	0.011	0.076**	0.011	-	-
4	23.643**	1.252	0.222**	0.018	0.271**	0.019	-	+
5	4.273**	0.739	0.036**	0.011	0.382**	0.011	-	-
Salmonella								
1	97.618**	0.286	0.003	0.004	-0.008	0.004	-	-
2	80.521**	0.905	0.139**	0.013	0.044*	0.013	-	-
3	60.649**	0.928	0.258**	0.014	0.065**	0.014	-	-
4	19.890**	1.184	0.153**	0.017	0.328**	0.017	-	-
5	8.584**	0.924	0.022**	0.014	0.431**	0.014	-	-
Clostridium								
1	98.521**	0.416	-0.002	0.006	0.001	0.006	-	-
2	94.779**	1.069	0.197**	0.016	-0.012	0.016	-	-
3	73.744**	1.174	0.347**	0.017	0.065**	0.017	-	-
4	29.401**	1.791	0.221**	0.026	0.417**	0.026	-	-
5	1.803	1.13	0.019	0.016	0.445**	0.016	-	-
Unknown Etiology								
1	94.840**	0.321	-0.023**	0.005	0.032**	0.005	-	-
2	83.812**	0.626	0.185**	0.009	0.070**	0.009	-	-
3	57.375**	0.756	0.348**	0.011	0.081**	0.011	-	-
4	21.036**	1.221	0.193**	0.018	0.243**	0.018	-	+
5	4.751**	0.613	0.048**	0.009	0.295**	0.009	-	-
Multiple Etiology								
1	98.533**	0.447	-0.001	0.007	-0.007	0.006	-	-
2	90.925**	1.743	0.124**	0.026	-0.018	0.024	-	-
3	70.290**	1.576	0.342**	0.024	0.037	0.022	-	-
4	20.769**	1.974	0.176**	0.030	0.365**	0.027	-	-
5	2.237	1.284	0.018	0.019	0.502**	0.018	-	-

Estimation with ** represents p-value <0.001 and * represents p-value<0.05. Seasonality with plus sign indicates a seasonal pattern was detected while a minus sign indicates no seasonal pattern .

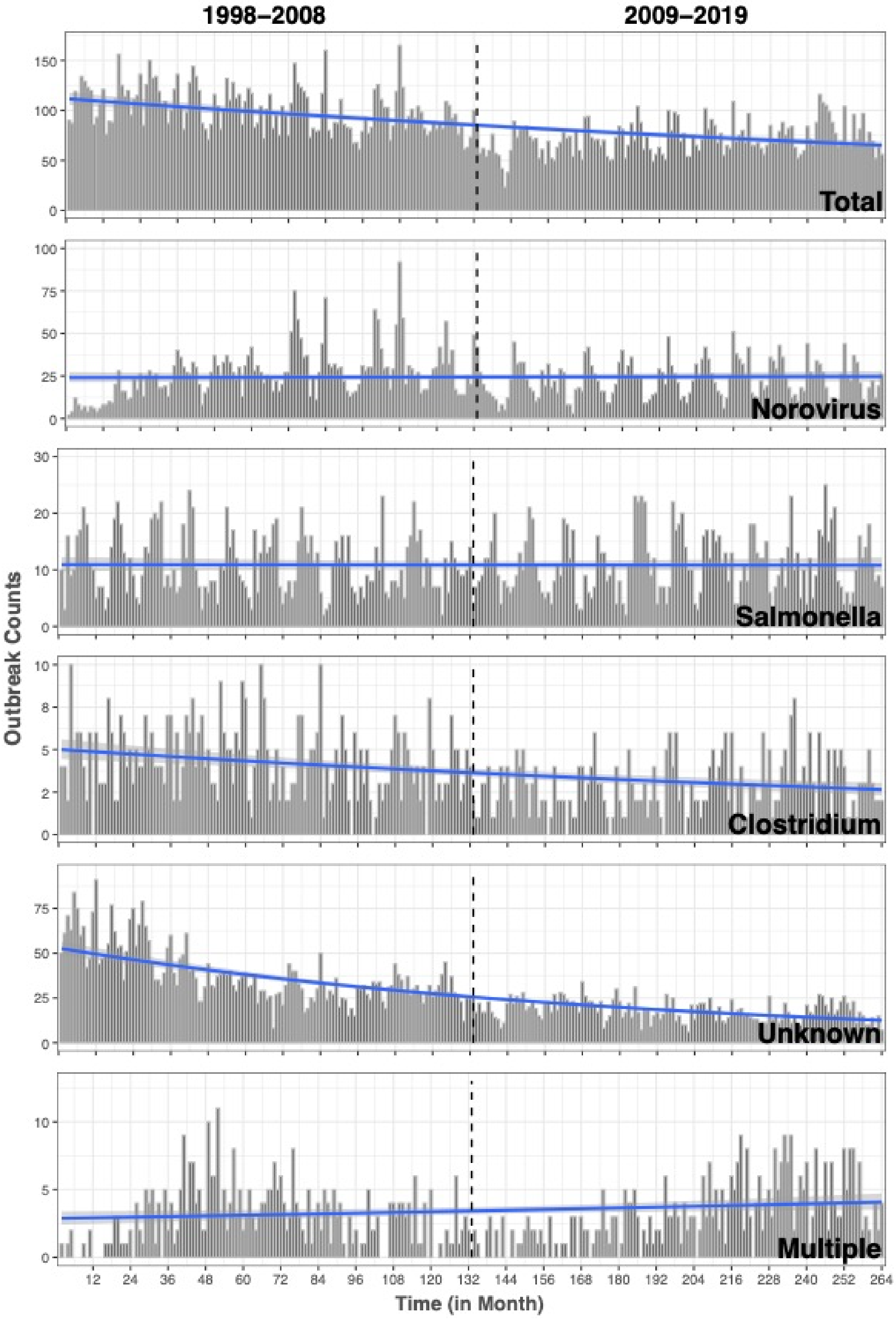

Supplementary Figure S1. The number of foodborne outbreaks per month reported in NORS from 1998 to 2019 (gray bars) with fitted negative binomial regression (Model 1, blue line). The left-axis is the number of outbreaks reported in each month in the pathogen group. The bottom-axis is the month associated with the outbreak with 1 being January 1998 and 264 being December 2019.

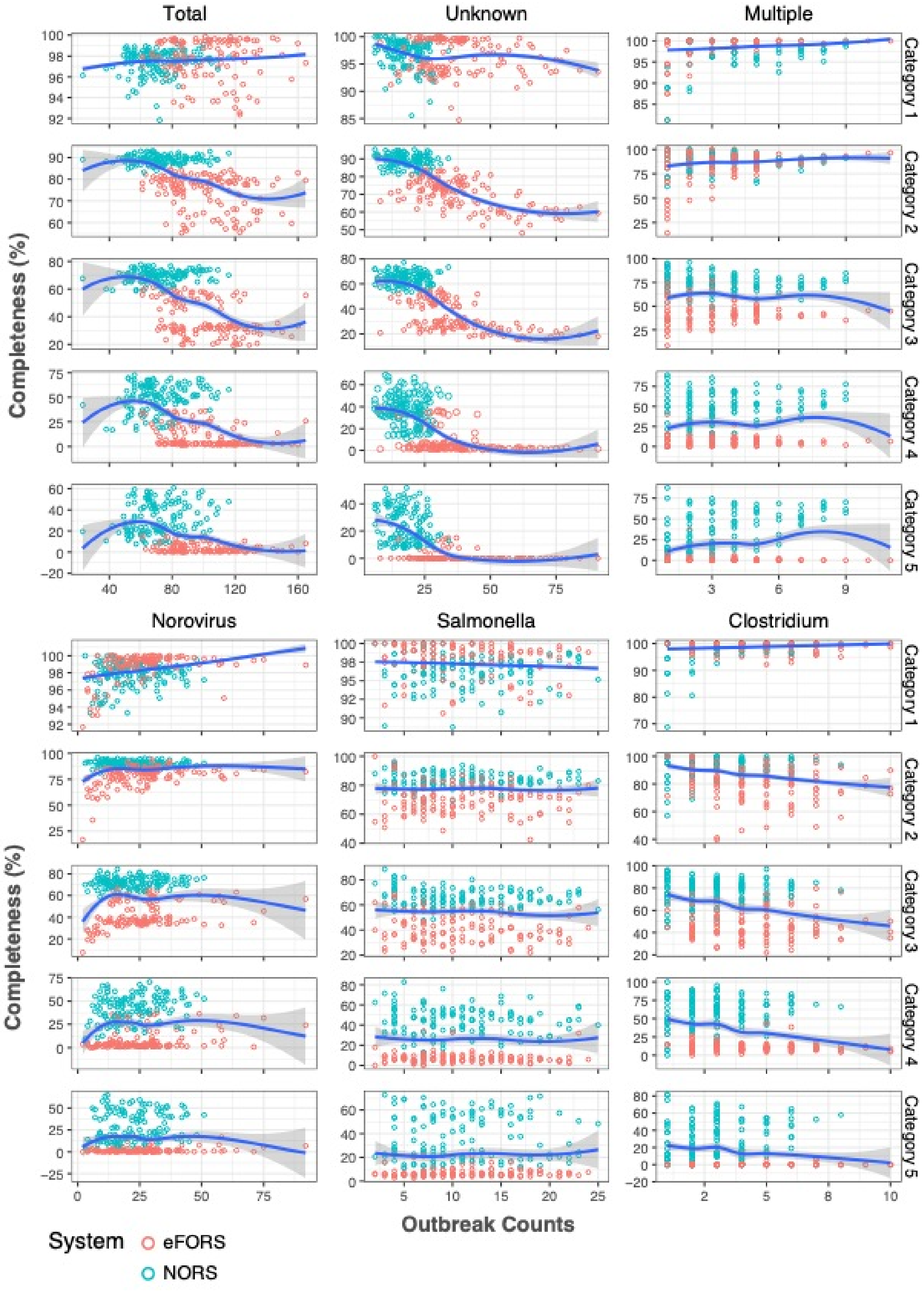

Supplementary Figure S2. Outbreak count per month in relation to average completeness in each category using overall outbreak data and per pathogen group presented as loess-smoothed curves (Model 2, blue curve) along with the 95% confidence interval. The scales in each graph are adopted to their data range. The orange color indicates eFORS data and blue color indicates NORS data.

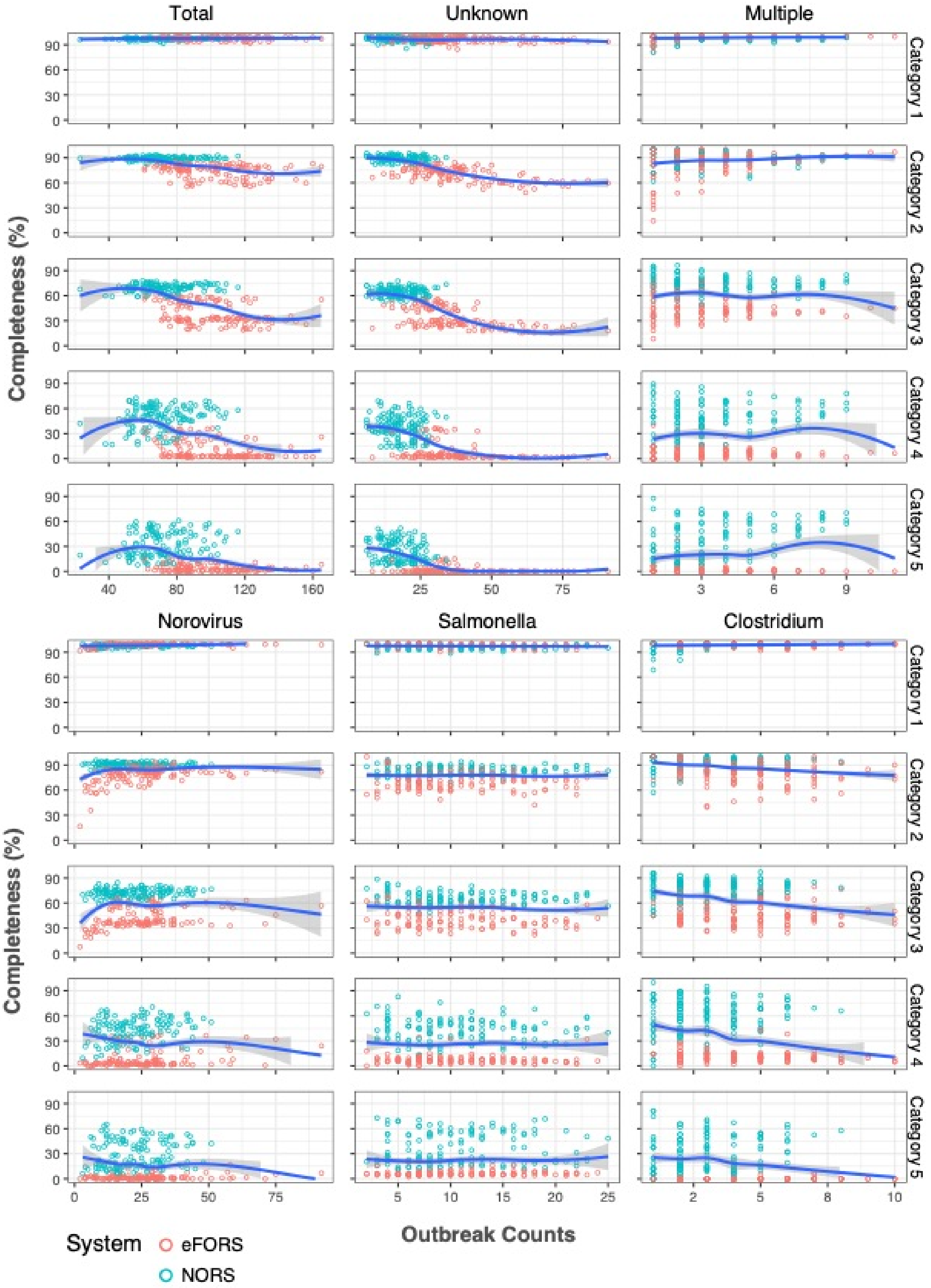

Supplementary Figure S3. Equally scaled outbreak count per month in relation to average completeness in each category using overall outbreak data and per pathogen group presented as loess-smoothed curves (Model 2, blue curve) along with the 95% confidence interval. This figure is a duplication of Figure S2 with the same 0-100 scale in left-axis across all categories and pathogen types, aiming for comparable visualization of the trends among different categories. The orange color indicates eFORS data and blue color indicates NORS data.

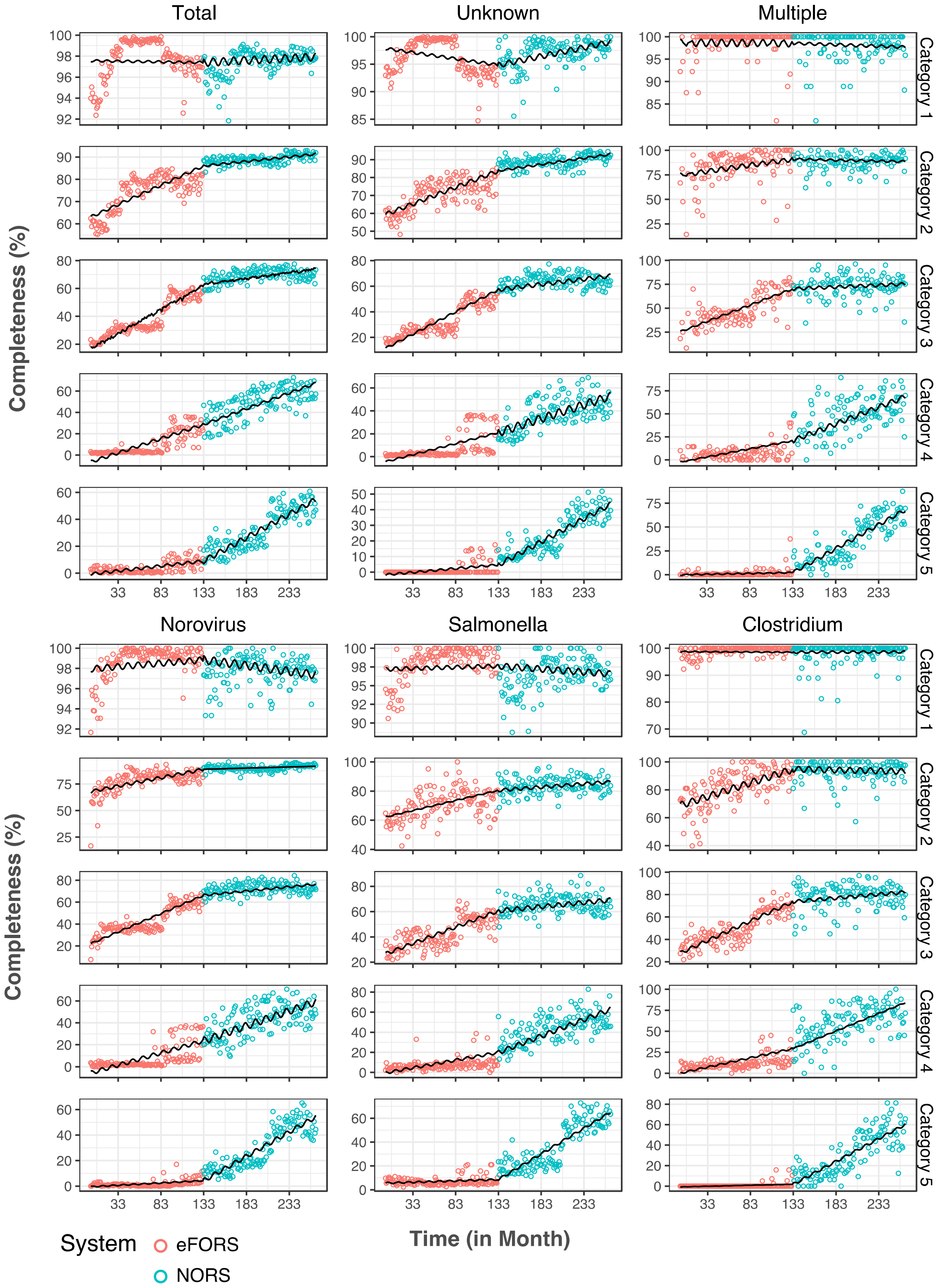

Supplementary Figure S4. Seasonality analysis for overall data and per pathogen group in each category. The black curves represent the predicted completeness (fitted from Model 3). The vertical-axis represents the average completeness in each category by month and the bottom-axis represents month (e.g., 0 = January 1998, 133 = January 2009, and 264 = December 2019). The scales in each graph are adopted to their data range. The orange color indicates eFORS data and blue color indicates NORS data.

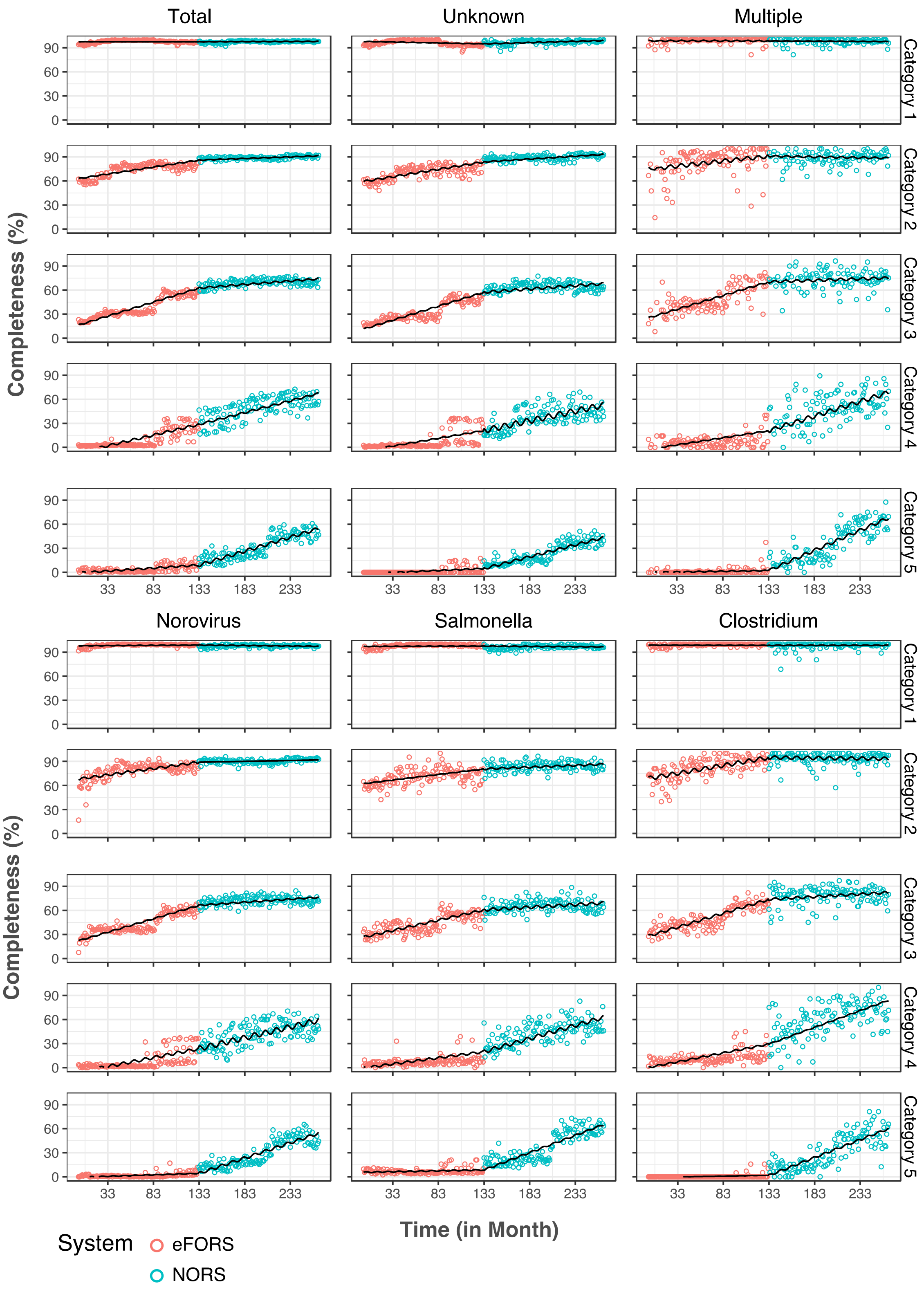

Supplementary Figure S5. Equally scaled seasonality analysis for overall data and per pathogen group in each category. The black curves represent the predicted completeness (fitted from Model 3). The vertical axis represents the average completeness in each category by month in same scale 0-100%. The bottom-axis represents month (e.g., 0 = January 1998, 133 = January 2009, and 264 = December 2019). The orange color indicates eFORS data and blue color indicates NORS data.
